# The Active Role of Leguminous Plant Components in Type 2 Diabetes

**DOI:** 10.1155/2014/293961

**Published:** 2014-03-11

**Authors:** Monika Gętek, Natalia Czech, Małgorzata Muc-Wierzgoń, Elżbieta Grochowska-Niedworok, Teresa Kokot, Ewa Nowakowska-Zajdel

**Affiliations:** ^1^Department of Internal Medicine, Bytom, Silesian Medical University, 41-902 Bytom, Katowice, Poland; ^2^Department of Human Nutrition, Zabrze, Silesian Medical University, 41-808 Zabrze, Katowice, Poland

## Abstract

Diabetes appears to be one of the most frequent noncommunicable diseases in the world. A permanent growth in the incidence of diabetes can be observed and according to the International Diabetes Federation (IDF) the year 2030 will mark the increase in the number of diabetics to 439 mln worldwide. Type 2 diabetes accounts for about 90% of all diabetes incidence. Nutrition model modification not only features the basic element in type 2 diabetes treatment but also constitutes the fundamental factor influencing a morbidity rate decrease. Leguminous plants are a key factor in the diabetic diet; plants such as pulses or soybeans are nutritious products valued highly in nutrition. These legumes are high in the content of wholesome protein and contain large amounts of soluble alimentary fiber fractions, polyunsaturated fatty acids, vitamins and minerals, and bioactive substances with antioxidant, anti-inflammatory, and anticancer activity. They are distinguished by the high amount of bioactive compounds that may interfere with the metabolism of glucose. The most significant bioactive compounds displaying antidiabetic activity in leguminous plants are as follows: genistein and daidzein, alpha-amylase inhibitors, and alpha-glucosidase inhibitors. *In vitro* research using leguminous plant extracts has confirmed their antidiabetic properties. Leguminous plants should be employed in the promotion of healthy lifestyles in terms of functional food.

## 1. Introduction

Diabetes appears to be one of the most frequent noncommunicable diseases in the world. A permanent growth in the incidence of diabetes can be observed and according to the International Diabetes Federation (IDF) the year 2030 will mark the increase in the number of diabetics to 439 mln worldwide. In Europe, the highest morbidity rates are observed in Germany (8.9% population), Spain (8.7%), and Belgium (8.0%) and the lowest rates in Great Britain (4.9%) and Sweden (5.2%). The biggest populations of diabetics worldwide, however, are to be found in India (51 mln), China (43 mln), and the USA (27 mln).

Type 2 diabetes accounts for about 90% of all diabetes incidence. It is estimated that about 50% of diabetics remain undiagnosed [1]. Judging from epidemiological research, the most important hazard factors leading to type 2 diabetes are as follows: obesity, arterial hypertension, lipid balance disorder, highly calorific diet with a prevalence of saturated fats, limited physical activity, ageing processes, and tobacco smoking [2–4]. In type 2 diabetes pathogenesis a significant influence is caused by hypoinsulinaemia resulting from *β* cell pancreatic islet secretory functional disorder together with lowered peripheral tissue sensitivity to insulin activity (fatty tissue, muscles, liver, and others). Both growing insulin resistance and hyperinsulinism are observed because a decline in sensitivity to insulin leads to enhanced secretion of this hormone before the first meal, that is, on an empty stomach and after the meal. Nutrition model modification constitutes not only the basic element in type 2 diabetes treatment but also the fundamental factor influencing a morbidity rate decrease. Modifying nutrition patterns involves a volume reduction in easily absorbed carbohydrates in favour of low glycaemia products, an increase in the daily supply of nutrifiber, and a reduction of fatty product uptake in the overall daily energy supply. This is especially true for saturated fats while increasing the amount of polyunsaturated fatty acids.

Leguminous plants, for example, pulses (dry beans, chickpeas, lentils, and peas) and oil seed (soybeans), are a key factor in the diabetic diet. These plants are nutritious products valued highly in nutrition. This specific group provides wholesome products of vegetable protein whose volume ranges from 20% in beans and peas up to 38–40% in soya beans. These proteins contain a large amount of lysine, especially in beans, which is the reason they are regarded as wholesome. Protein nutritive value is reduced by subdued sensitivity to proteolysis, low contents of sulphur amino acids, and the presence of nonprotein substances such as phytic acid and tannic acid and nonphysiological proteins (lactinine, protease inhibitors). The legumes are known to contain large amount of soluble alimentary fiber fractions (4–6%), polyunsaturated fatty acids (18% in soya bean), B group vitamins, and minerals (phosphorus, potassium, calcium, magnesium, iron, zinc, and copper). They are described as possessing a low glycaemia index (<50) and they are alkalogenic products, which is especially vital in acid-alkaline balance maintenance in organisms [5–7]. Leguminous plants are notable for their high levels of bioactive compounds, which can influence glucose metabolism by the following:

1. carbohydrate digestion inhibition and the suppression of glucose absorption in the intestine,
2. stimulation of insulin secretion from pancreatic *β* cell liver glucose release modulation,
3. insulin receptor activation [8].

Legume intake worldwide differs depending on the region. The highest intakes are registered in South America (10.7 kg/person annually), Africa (9.8 kg/person annually), and Asia (5.9 kg/person annually) [5]. The average consumption in Europe comes to about 2.7 kg/person annually (FAOSTAT 2009). In Poland it is round 1 kg/person annually and at the end of the last decade it fell by 43% [9].

## 2. Bioactive Substances Contained in Leguminous Plants

The most crucial bioactive compounds displaying antidiabetic activity in leguminous plants are as follows:

1. genistein and daidzein,
2. alpha-amylase inhibitors,
3. alpha-glucosidase inhibitors.

Genistein and daidzein are natural estrogens found in soya beans and soya derivative products. Their similarity to estrogens enables binding to receptors in human cells and in choriocarcinoma cell lines as well (BeWo and Jeg3) [10]. High dosages of genistein and daidzein may reduce cell proliferation and the production of the steroid hormones as progesterone [10]. Natural estrogens are able to bind to both estrogen receptors in humans (ER*α* and ER*β*) but with a higher affinity to ER*β* [11]. Genistein has been shown to have a 20-fold higher binding affinity to ER*β* than ER*α* and when compared to 17*β* estradiol affinity for ER*α*, the potency of genistein for the receptor is 130-fold lower [12, 13]. Genistein displays both agonist and antagonist activity towards the estrogenic receptors which may be important in breast cancer prevention [14]. A similar activity is manifested by tamoxifen which is applied in breast cancer treatment. Estrogen equol and genistein can replace natural estrogen and testosterone. These isoflavonoids influence the reproduction, growth, and maturation of cells. They perform important regulatory functions in the maintenance of organ activity. As antioxidants, genistein and daidzein protect cells against the effects of harmful free radicals which are responsible for ageing and concomitant diseases such as atherosclerosis, arthritis, or diabetes [15, 16].

Equol is a metabolite of soy isoflavones—daidzin and daidzein. It is produced by intestinal bacteria but not in all people. The term equol-producers identifies those individuals who can produce equol in response to consumption of soy isoflavones. There is also an established hypothesis that equol-producers consuming soy diet had greater health benefits than equol-nonproducers [17].

The alpha-amylases inhibitors are, inter alia, compounds which suppress carbohydrate absorption through inhibiting enzymes responsible for starch digestion. Alpha-amylase inhibitors are found in legumes, fruit, green and black teas, wheat, and rice. Three isoforms of alpha-amylase inhibitors are found in legumes: alpha-amylase inhibitor isoform 1 (Alpha- AI1), alpha-amylase inhibitor isoform 2 (Alpha-A12), and alpha-amylase inhibitor isoform like (Alpha-AIL). The alpha-AI1 isoform displays activity in humans. Phaseolamin is the generic name for the common white bean extract containing alpha-amylase inhibitors. Alpha-AI1 is found in germs and seeds and cannot be found in other plant parts. Alpha-AI1 synthesis occurs at the same time as the phaseoline and phytohaemagglutinin (PHA), stored in vacuole. These are typical lectines, synthesised in rough endoplasmic reticulum and modified through Golgi apparatus in the form of signal peptide removal and N-glycosylation. They possess homologous amino acid sequences, from 50 to 90% [18, 19].

Alpha-AI1 is detected 17 days after pollination in seed axes and cotyledons. Regular growth happens up till the 28th day. The level of Alpha-AI1 decreases slightly during the drying process [18]. Alpha-AI1 prevents starch digestion through blocking complete access to the active enzyme position. The factors which refer to Alpha-AI1 inhibitor activity are reaction, temperature, incubation period, and certain ion presence. The optimal reaction pH value for the inhibitor is 4.5–5.5 and optimal temperature is 22–37°C, and it becomes inactivated completely after 10 minutes of cooking [20].

*In vitro* research has proved the inhibiting effect of assorted bean variety extracts on alpha-glycosidase, especially in the case of “Azuki” beans. However, it was Yao et al. who described the bioactive components—flavonoids responsible for this effect. These are vitexin and isovitexin. Vitexin activity (apigenin C glucoside) thus far confirmed concerns the widening of coronary vessels, angina pectoris prevention, sedative functions, and spasmolytic and anti-inflammatory activity. Vitexin is pharmacologically significant due to its ability to improve coronary flow. It is listed as a mild cardiac drug. Isovitexin (2-0-GIV) makes a strong antioxidant. The blocking effect of these flavonoids on alpha-glycosidase is also confirmed by research conducted on other plants [21–23] (see Table 1).

## 3. Genistein and Daidzein: Antidiabetic Activity

### 3.1. Animal Studies

Genistein and daidzein are two of the the best known flavonoids which have effects on secretory pancreas functions. Research confirms that these substances in physiologically achieved concentrations exert beneficial effects on the functions of pancreatic *β* cells [48].

Choi et al. tested the genistein and daidzein influence on glucose and insulin metabolism in nonfat mice, in which autoimmunological, insulin-dependent diabetes was developed [29]. Isoflavonoids supplied at the dosage of 0.2 g/kg within nine weeks led to insulin production maintenance by pancreatic *β* cells, while with mice from the control group insulin production did not appear [29]. Another examination* in vivo* carried out on nonfat mice with STZ-induced diabetes, fed with fermented soya (5 g genistein/100 g 6 week-diet), proved that insulin doses in pancreas were higher with the soya-fed mice group in comparison to the control one. Apart from increases in pancreatic insulin production, soya dieting contributed to an improvement in peripheral tissue sensitivity to insulin [30]. Similar results were achieved by Lu et al. testing rats with STZ-induced diabetes and fed on a highly isoflavonic diet [48].

Soya isoflavonoids also display the regulatory ability of triglyceride synthesis in the liver. According to the mentioned examination by Lu et al. carried out on nonobese, diabetic mice, soya isoflavonoids, genistein, and daidzein were applied at the ratio of 0.2 mg/kg lower glucose levels in blood thus decreasing triglyceride gradients in the liver. The research corroborated diminishing glucose-6-phosphatase activity and phosphoenolpyruvate carboxykinase (PEPCK) as well, together with a glucokinase activity increase, suggesting that genistein and daidzein block glucose production in the liver [29, 30].

Cederroth et al. tested the effect of daidzein and genistein supplementation in healthy mice CD-1. As a result of a phytoestrogen rich diet (198 ppm daidzein and 286 ppm genistein) from the conception period up to adult age, AMP-activated protein kinase (AMPK) activation was observed in the liver and in white fatty tissue, as well as in muscles. This includes reduced lipid contents in adipocytes, increased phosphorylation of AMPK and acetyl-CoA carboxylase (ACC), increased expression of peroxisome proliferator-activated receptor gamma coactivator (PPAR-l*γ*) and genes involved in mitochondrial biogenesis and reactive oxygen-species detoxification (ROS) enzymes, increased insulin sensitivity, and reduction of the pathway mammalian target of rapamycin/ribosomal protein S6 kinase beta-1 (mTOR/S6K1) in skeletal muscle. It was discovered that a high phytoestrogen diet may alter mitochondrial metabolism and disturb expression of peroxisome proliferator activated receptor alpha (PPAR*α*) and PPAR-l*γ* in the liver, white adipose tissue, and muscles suggesting fatty-acids beta-oxidation [31]. Potentially, with nonfatty mice PPAR*α* activation may lead to a change in glucose utilization into fatty acid oxidation instead of new triglyceride progression [49]. Increased fatty acid oxidation may protect against nonalcoholic liver steatosis and improve sensitivity to insulin and glucose metabolism in the liver. Moreover, a decrease in liver triglycerides is correlated with lowered insulin resistance; however, the governing mechanism of triglyceride influence on insulin resistance has not been recognized [50].

Kim and Lim studied diabetic mice which were divided into two groups according to fasting glucose levels (FBG): medium high FBG (DMMH; FBG 250–450 mg/dL) and high FBG (DMH; FBG 450–600 mg/dL). Mice were fed with different diets and further divided into the following groups (*n* = 9 or 10 individuals in each group): nondiabetic mice (CON), diabetic mice in the control group (DMC; DMMH-C, DMH-C) fed without genistein supplementation, and diabetic mice with genistein supplementation: DMMH—0.025%, DMH—0.025%, DMMH—0.1%, and DMH—0.1%. At the end of treatment body weight, food intake, and fasting blood glucose level were measured once a week. Genistein supplementation did not prevent the decrease of body weight. Food intake is significantly increased in diabetic mice with genistein supplementation compared to the CON group. Only 0.025% genistein supplementation in DMMH significantly reduced the level of FBG. Genistein supplementation in the DMH is not reflected in a difference in the levels of FBG. Plasma concentrations of total cholesterol and triglyceride were more elevated in DMC than in CON. There were no significant differences between DMC and the groups of genistein supplementation. Plasma levels of high density lipoprotein cholesterol did not differ between the groups [43].

Fu et al. tested 32 male C57BL/6 mice with obesity and diabetes generated by the high fat content of the diet and low dosage streptozotocin injections. The mice were divided into 4 groups (8 mice in each group) and fed a standard diet with 10% of calories from fat, a diet with a high fat content of 60% of calories from fat, and a diet containing 250 mg genistein/kg body weight. Consumption of genistein (250 mg/kg (−1)) resulted in improved hyperglycaemia, glucose tolerance, and insulin levels in the blood. This diet did not affect body weight gain, food intake, fat deposits, serum lipid profiles, or peripheral insulin sensitivity. Genistein caused an increase in the number of insulin-positive *β* cells of the islets, promoted survival of islet *β*, and kept islet mass [41].

Park et al. conducted research on male Slc: ICR mice in which diabetes was induced by injection of STZ, for the inhibitory effect of daidzein on alpha-amylase and alpha-glucosidase. Healthy mice and mice with STZ-induced diabetes were randomly divided into 3 groups of 7 mice. In the fasting state, the mice were orally administered soluble starch (2 g/kg) or daidzein, starch (10 mg/kg) or acarbose, and starch (10 mg/kg). The effect of daidzein in postprandial blood glucose levels was studied in normal and diabetic mice. Blood glucose levels in mice treated with daidzein were lower than in both the control mice and mice fed a diet supplemented with acarbose. When daidzein was added to healthy mice diet, the increase in postprandial blood glucose levels was significantly inhibited (16.79 ± 2.08, 18.72 ± 1.03, and 15.37 ± 2.10 mmol in 30, 60, and 120 minutes, respectively, *P* ≤ 0.05). The postprandial blood glucose levels in healthy mice treated with daidzein starch were also significantly lower (*P* ≤ 0.05). Similarly, the area under the curve of blood glucose levels in the diabetic mice group fed with starch and daidzein (2043.0 ± 204.9 mmol min L) was significantly lower (*P* ≤ 0.05) than the control group (204.2 ± 2475.2 mmol min L) [45].

*In vitro* studies indicate both the effect of (S)-equol to activate PPAR and the elevation in the expression of GLUT4 and IRS-1 by which in turn suspends insulin-stimulated glucose uptake in adipocytes. Other* in vitro* studies have shown that (S)-equol affects rat gene SHARP 2. The conclusions of both studies indicate that (S)-equol and daidzein may be useful in the control of type 2 diabetes [51, 52].

### 3.2. Clinical Studies

Clinical studies show conflicting results.

Genistein does not affect glucose transporter-2 expression or adenosine triphosphate (ATP) cellular production but increases pyruvate-stimulated insulin secretion in INS-1E cells, which indicates that insulin secretory function improvement through long-lasting exposure to genistein is not connected with alternative glucose uptake or glycolysis pathways. Increased insulin secretion due to genistein is connected with increased intracellular Ca^2+^ ion gradient and is protein kinase A dependent together with new proteins synthesis, and this effect is utterly blocked by N-[2-(p-bromocinnamylamino)ethyl]-5 or isoquinoline sulphonamide cycloheximide. These results prove that genistein may be a new bioactive compound displaying antidiabetic effects owing to pancreatic *β* cell insulin secretion improvement [32].

In a randomized study of a 12-month, double-blind trial, a placebo controlled trial was attended by 120 women with metabolic syndrome, aged 49–67 years, and postmenopausal for at least 12 months. They received either genistein (*n* = 60), 54 mg/day, or a placebo (*n* = 60). Genistein in serum increased after 6 and 12 months only in patients in the group treated with genistein. In this group glucose and insulin levels were significantly decreased. These two parameters did not change in the placebo group. Horizontal total cholesterol and triglyceride decreased statistically at both time points compared to the placebo. HDL levels increased significantly in the group treated with genistein. In the placebo group there was no change. Systolic and diastolic blood pressure were significantly reduced in the group receiving genistein. BMI showed no significant changes in both groups. Visfatin and homocysteine levels were significantly reduced at 6 and 12 months of the study only in the group receiving genistein [42].

In a randomized, double-blind, placebo-controlled group, Gobert et al. studied the effects of soy protein and isoflavones and did not receive a positive result. Adults with type 2 diabetes (*n* = 29) consuming a diet containing soy protein (SPI) and milk protein isolate (MPI) were studied. The study lasted 57 days. Periods of dietary SPI and MPI were separated by 4 weeks of washout. Blood samples were collected on days 1 and 57 of each treatment period for analysis of fasting HbA1c, postprandial and fasting plasma glucose, and a fasting serum insulin level and the calculated ratios and sensitivity to insulin resistance. In order to analyse isoflavone in urine, samples were collected 24 hours after the end of each treatment period. Isolates were comparable to the following energy and nutrient contents per day: 837 kJ, 8-9 grams of carbohydrates, 40 g protein from soy (SPI) or milk (MPI), 88 mg (SPI), or 0 mg (MPI) isoflavones (65% genistein, 31% daidzein, and 4% glycitein). Isoflavone excretion was significantly higher in the SPI group compared with the MPI. 20.7% of patients (*n* = 6) were classified as equol excretors. Consumption of the SPI diet did not significantly influence the level of fasting and postprandial glucose, insulin, fasting HbA1c, and indexes of insulin sensitivity and resistance.

When the authors considered the analysis of equol excretors as a variable in the statistical model the results were not changed and there was no significant interaction between equol production by individuals and diet treatment without the markers of glycemic control [36].

## 4. Alpha-Amylase Inhibitors

Proteins are one of the basic components of food but have different nutritional values because they contain very different amino acid compositions. The value is determined by the number of amino acids of proteins, their qualitative composition, bioavailability, and digestibility. Proteins ensure proper construction of tissue, regulate metabolic processes, and facilitate the absorption of minerals. Peptides derived from food proteins affect the circulatory system, nervous system, alimentary system, immune system, and functional properties. One of several activities is regulation of enzyme inhibitory peptides. At the cellular level proteins can act by inactivating ribosomes or inhibiting the active site cell, such as alpha-amylase inhibitors [53].

The monitoring of carbohydrate digestion as well as glucose absorption enables better control of postprandial glycaemia, especially after highly carbohydratic meals. Released glucose is absorbed by intestinal enterocytes through transmitters. Digestive enzyme inhibition or glucose transporters reduce glucose release and absorption in the small intestine and, as a consequence, suppress postprandial hyperglycaemia.

In the* in vitro* research it was proved that white beans bind alpha-amylase forming a 1 : 1 complex blocking the enzyme. The research on rats showed that it decreases basic and postprandial glucose levels in blood. Moreover, it diminishes appetite, which, in turn, contributes to weight control [54].

In three experiments on healthy Wistar rats, Loi et al. studied the effect on hypoglycaemia and decreasing food intake of* Phaseolus vulgaris* extract (PVE) and* Cynara scolymus* extract (CSE). In experiment 1, 36 rats were divided into 4 groups (*n* = 9). Three days before the experiment, rats were treated acutely with three different compositions of PVE, CSE, PVE+CSE, or without extracts. In experiment 2, 32 rats were used. Rats were fed a chocolate drink, a normal chow diet, and water. After 15 days feeding rats were divided into 4 groups (*n* = 8) and treated acutely for 3 days like the previous group. In experiment 3, 28 rats, after 6-hour fasting, were divided into 4 groups (*n* = 7) of three different compositions of PVE, CSE, PVE+CSE (the doses were different compared to groups 1 and 2), or without extracts. When the lights in the cages were off rats were fed 3 g/kg starch. In experiment 1, administration compositions which included only PVE or PVE+CSE showed a statistically significant effect in lowering blood glucose and consumption over 24 hours (*P* ≤ 0.0001). There was a reduction of food intake by an average of 25%. In experiment 2, after consuming a chocolate beverage on the test day a decrease in blood glucose and food intake was noted in the PVE and PVE+CSE groups compared to the control group (*P* ≤ 0.0005). The reduction of food intake was on average 35%. As a result of experiment 3 the PVE+CSE (*P* ≤ 0.005) group showed a statistically significant decrease in blood glucose levels to 33% [44].

White bean extract, apart from alpha-amylase inhibition, stimulates cholecystokinin (CCK) and disturbs the food uptake mechanism. A high ratio of phaseolamin application weakens rat growth speed [55]. The research conducted on people showed contradictory results.

Udani et al. tested the influence of water extract of a common white bean on weight loss and triglyceride level. The randomized examination was carried out using a double-blank test on a panel of 39 obese people (35 women and 4 men) who were given 1500 mg water extract of a common white bean with their meals, twice a day. The examination lasted 8 weeks. The results showed a higher weight loss and triglyceride level decrease in the group obtaining the extract. The differences were not statistically significant [24]. Another control examination carried out among 10 participants with normoglycaemia proved that phaseolamin caused a quicker postprandial glycaemia reduction than in the control group, and glucose absorption was 57% lower [56].

In a randomized, double-blind, placebo-controlled study examining 60 overweight (5–15 kg above) people, Celleno et al. showed that the use of Phase 2 results in a statistically significant decrease in body weight, fat mass, and WHR. Patients received either one 445 mg tablet of Phase 2 + 0.5 mg chromium picolinate or a placebo daily for 30 consecutive days prior to a meal rich in carbohydrates (2000–2200 calories). Body weight, BMI, fat mass, adipose tissue thickness, and waist,/hip/ thigh circumferences were reduced after 30 days [26].

Wu et al. in a randomized, double-blind, and placebo-controlled study examined 101 volunteers ( 50 placebo group and 51 study group) with a BMI between 25 and 40. The study group received two 1000 mg Phase 2 capsules or a reference substance in the form of two capsules containing microcrystalline cellulose of a volume and appearance of the capsules of Phase 2. Subjects treated with Phase 2 showed a statistically significantly greater decrease in both body weight 1.9 kg compared to 0.4 kg in the placebo group and waist circumference 1.9 cm compared to 0.4 cm. Accordingly, body weight and BMI dropped significantly too. There was no significant change in hip circumference. Among the biochemical parameters, only the creatinine value was higher and GPT lower in the group receiving Phase 2; however, the differences were not statistically significant [35].

In further studies, the authors obtained statistically significant results but the tested groups were not very large.

Udani and Singh research used a randomized, double-blank test method, which lasted 4 weeks and involved 25 healthy participants. Twice a day the tested subjects consumed 1000 mg fractionated white bean PVE or placebo between meals. All the tested participants were additionally subjected to a weight-loss plan which was based on a diet (some participants consumed high-carbohydratic meals) and exercises. In both groups, that obtaining the extract and that receiving the placebo, weight loss was observed. A division was made in respect to the amount of consumed carbohydrates. In the group placed in the highest tertile of carbohydrate uptake, the tested panel obtaining fractionated white bean PVE, there was a significantly bigger weight loss together with fatty mass decrease compared to the placebo group (*P* = 0.042) [25].

Udani et al. again tested Phase 2 impact on blood glucose levels in conducted open-label, crossover, 6-arm, randomized study of 13 people with a BMI of 18–25. This time the authors wanted to determine whether the addition of Phase 2 of high-GI foods (white bread) lowers GI. They carried out GI standardized tests by measuring blood glucose concentrations after consumption of white bread with butter, with and without the addition of Phase 2 in the form of capsules or powder. In both preparations, Phase 2 was administered at dosages up to 1500 mg, 2000 mg, and 3000 mg. Phase 2 powder was mixed with butter. The dosage of 2000 mg and 3000 mg Phase 2 capsules resulted in a slight reduction in GI. The dosage of 1500 mg and 2000 mg of Phase 2 powder caused a slight reduction in GI. A significant result was obtained with dosage of 3000 mg Phase 2 powder which is about 34.11% reduction in blood glucose levels after a meal (*P* = 0.023) [34].

Vinson et al. obtained similar results to Udani et al. when they conducted 2 studies, a crossover study and a placebo-controlled one. The first study involved 11 respondents (men and women, aged 21 to 57). Subjects were given 4 slices of white bread and 42 g margarine supplemented or not with 1500 mg of Phase 2. Phase 2 was added to the margarine. The meals were 610 calories and 60.5 g carbohydrates. Tests were made one week apart measuring glucose concentrations in blood plasma. In the study group average plasma glucose concentration versus the time curve was 66% lower compared to the control group (*P* ≤ 0.05). It is concluded that the addition of 1500 mg of Phase 2 to the meal will absorb 1/3 carbohydrates from white bread. In the second study with 7 patients (men and women aged 23–43 years) the authors tested another lower dosage of Phase 2: 750 mg. Members had previously eaten frozen meals consisting of fried steak, mashed potatoes, green beans, and cherry-apple pie with or without 750 mg of Phase 2 (mixed with the sauce). The meals were 630 calories and 64 g of carbohydrates. The study decreased average plasma glucose concentrations versus time curve by 28%. It is concluded that 2/3 of carbohydrates from the meal were absorbed. It can be seen from the result of the dose dependency [33].

Spadafranca et al., as with previous authors, studied the impact of PVE on the levels of glucose and insulin, C-peptide concentrations in blood plasma, and ghrelin after a meal and the impact on the control of appetite. A double-blind, randomized, crossover study included 12 healthy subjects (6 women, 6 men) aged 20–26 years, with normal weight (BMI: 19.7–23.5, fat: 31.5–11.5%). The subjects, in fasting state for at least 12 hours, received standardized meals consisting of a sandwich of white bread, ham, butter, and tomato +100 mg tablets of PVE (≥6% tested alpha-amylase inhibitor and phytohemagglutinin) or 100 mg placebo tablets. PVE or placebo tablets were consumed just before a meal. After a 7-day washout period the test was repeated. PVE supplementation reduced postprandial glucose, insulin, and C-peptide excursions and affected satiety sensations, inducing a lower desire to eat [39].

## 5. Alpha-Glucosidase Inhibitors

Alpha-glucosidase generated by the small intestine epithelium is one of the key enzymes responsible for carbohydrate digestion and triglyceride absorption. Its function is one of the modulating factors of postprandial hyperglycaemia. Intestinal alpha-glucosidase activity suppressors, just like alpha-amylase, may delay the digestion process and carbohydrate absorption and lower postprandial hyperglycaemia [8, 57].

“Azuki” beans underwent thorough research into their biological activity. In Asia, especially in Japan, Korea, and China, this food is recommended as suitable for diabetic patients owing to its high alimentary fibre contents as well as protein [58]. The latest research has confirmed large amounts of bioactive components in its contents, including phenol compounds [59]. Itoh et al. proved that “Azuki” beans display alpha-glucosidase enzyme blocking activity in rats with STZ-induced diabetes. It has also been observed that “Azuki” manifests the highest ability to block this enzyme among sixteen other bean variations. Research on antidiabetic “Azuki” bean property has focused mainly on bean extract activity, and its components have not been defined strictly [60].

Sreerama et al. in their research conducted on extracts of four-bean variations (mung, moth, red, and black “Azuki” variations) confirmed the biggest influence in terms of alpha-glucosidase blocking in the black “Azuki” bean variation. Mung beans showed the least activity [57].

In their research, Yao et al. for the first time ascribed compounds present in “Azuki” beans which are active and display alpha-glucosidase enzyme blocking activity. Using 70% of ethanol, the authors extracted four fractions out of “Azuki” beans. The ethyl acetate (EtOAc) soluble fraction manifested the biggest alpha-glucosidase blocking activity. Two major active components, vitexin and isovitexin, were isolated from “Azuki” beans. Fluorescent spectroscopy examination corroborated these flavonoid properties. The most beneficial effect is achieved at the temperature of 37°C, in laboratory conditions [21].

Earlier authors have studied the antidiabetic effects of mung bean sprout extract (MBS: DCI, vitexin and isovitexin: 24.16, 11.68, 5.40 mg/g) and mung bean seed coat extract (MBSC: DCI, vitexin and isovitexin: 0.0053, 15.22 and 11.42 mg/g) using mice with type 2 diabetes: 50 KK-Ay male mice and 10 healthy male mice C57BL/6. Diabetic mice were divided into five groups and fed different dosages MBS (1, 2, 3 g/kg), MBSC (3 g/kg), and without extracts. Group 6 included C57BL/6 mice. For all mice the following were measured: glucose blood, the plasma C-peptide, glucagon, total cholesterol, triglycerides, and blood urea nitrogen (BUN). Oral administration of MBS (3 and 2 g/kg) and MBSC (3 g/kg) showed lowered blood glucose levels compared with control KK-Ay mice. The supplementation of MBS (2 g/kg) and MBSC (3 g/kg) reversed the blood C-peptide and glucagon levels in type 2 diabetic animals as compared with untreated diabetic mice. In mice fed with MBSC and 3 and 2 g/kg MBS increases in triglycerides and total cholesterol level were eliminated. The plasma levels of BUN in MBS groups (2 g/kg) were lowered as compared with the KK-Ay mice control group. Mice which have taken MBS (2 g/kg) and MBSC (3 g/kg) had a significant increase in insulin immunoreactivity as compared with untreated diabetic mice [28].

Choo et al. obtained positive results in healthy and diabetic animals, and they also investigated the inhibitory effect of isovitexin and vitexin from* Ficus deltoidea* for alpha-glucosidase.

Authors used healthy mice and rats with STZ-induced diabetes divided into 8 groups of 6 animals. Six groups of mice were dosed by oral gavage at three different dosages (1 mg/kg, 3 mg/kg, and 15 mg/kg) of vitexin or isovitexin. A positive control group of mice were given acarbose (3 mg/kg). Six groups of rats were dosed by oral gavage with various dosages of vitexin (50, 100, and 200 mg/kg) or isovitexin (20, 50, and 100 mg/kg). Positive control rats received acarbose (5 mg/kg). After administration of treatments to mice and rats, they were treated with sucrose at 2.5 g/kg and 4 g/kg, respectively. Normoglycemic mice given 1 mg/kg vitexin showed a significant (*P* ≤ 0.05) reduction in blood glucose levels by 24.7%. At higher dosages of 3 and 15 mg/kg, the percentage reduction increased to 26.5% and 31.3%. When mice were given 1, 3, and 15 mg/kg isovitexin blood glucose levels were significantly (*P* ≤ 0.05) lower at 30 minutes. The highest (19.7%) reduction in blood glucose level was observed in diabetic rats at 60 minutes when 200 mg/kg vitexin was administered orally. After administration of 100 mg/kg isovitexin the largest reduction in blood glucose level was observed (19.7%). The administration of all three doses of isovitexin significantly (*P* ≤ 0.05) reduced postprandial blood glucose concentrations [40].

The extrusion is the next method to obtain extracts from “Azuki” beans. This method decreases the antioxidant activity in extruded “Azuki” beans by 41.86% and increases the antidiabetic activity in inhibited rat intestinal alpha-glucosidase in extruded “Azuki” beans by 307.60% compared to raw “Azuki” beans.

Yao and Ren studied the antidiabetic effects of extruded “Azuki” beans on 80 male rats fasting. In 40 rats, diabetes was induced using 45 mg/kg STZ. Healthy and diabetic rats were divided into 4 groups each: control group, positive control group (50 mg/kg acarbose), extract “Azuki” bean group (ABE, 200 mg/kg), and extract extruded “Azuki” bean group (EABE, 200 mg/kg). 30 minutes after administration of treatment the animals were orally administered fasted sucrose (2 g/kg). In the control group of healthy rats, blood glucose levels averaged 10.4 mmol/L 30 minutes after the administration of sucrose. In the group treated with EABE the glucose level after 30 min only averaged 8.79 mmol/L j in comparison with the control group. Blood glucose levels dropped by 15.6% in the treatment with EABE and by 22.6% in the treatment with acarbose. In the control group of diabetic rats blood glucose levels rose to an average of 31.2 mmol/L 30 minutes after sucrose administration. However, the measurement of blood glucose levels in the groups that received EABE and acarbose was difficult to do. Compared with the control group, the glucose level decreased by 28.4% in the case of administration of 300 mg/kg EABE and 23.0% when administered with 20 mg/kg acarbose [46].

For comparison, Maruyama et al. tested “Azuki” bean extract activity on a panel of 33 female students. The mean age of the 33 subjects was 21.3 ± 0.8 years, BMI 19.8 ± 1.5, nonsmokers, without metabolic diseases, and with regular menstrual cycles, who had not used preparations with “Azuki” bean extract before. The chosen method was a double-blank test. The subjects were randomly divided into three groups: control, “Azuki,” and concentrated “Azuki” (CA) juice. All drank 150 g of the isocaloric assigned juice 5 times a day for one menstrual cycle, along with their staple diet uptake. The results showed that in the control group body weight went up slightly and triglyceride level was decreased substantially within the group consuming bean juice (*P* ≤ 0.05), whereas in the group taking CA, the triglycerides level was decreased statistically insignificantly (*P* = 0.055). The authors examined the alpha-glucosidase blocking faculty of “Azuki” juice and CA* in vitro*. A 100 mq dosage of bean extract reduced enzyme activity by 32.6% [27].

## 6. Conglutin Gamma Protein

Lupin plants, growing in popularity in Western Europe and Australia, are acknowledged alongside soya as one of the most valued vegetable protein sources. In* in vitro* research, using lupin isolated protein called conglutin gamma has proved to possess an insulin-binding property and to lower postprandial glycaemia. This activity has been compared to metformin [61]. In randomized research on 24 adult individuals it has been proved that adding lupin kernel flour with 12.5 g fiber and 22 g protein (lupin) into a drink containing 50 g glucose led to postprandial glycaemia reduction compared to subjects from a control group, consuming a drink with 50 g glucose added. Similar results were achieved in a group consuming a drink with 50 g glucose added plus 12.5 g fiber and 22 g protein from soya isolates (soya) [38].

Capraro et al. studied controlled body weight gain and hypoglycemic properties proteins derived from Lupin plants including conglutin gamma. Authors observed by 3 weeks, two groups of rats (fed pasta with or without addition of pure protein from Lupinus). In the results a significant reduction in food intake and glycaemia was observed in animals fed pasta with supplemented lupin protein [47].

In another experiment, Bertoglio et al. investigated the characteristics of dried extract rich in conglutin gamma (conglutin gamma content was about 47% of the total proteins in the dry powder) in rats, and for the first time in humans. The animal study was conducted on 100 rats divided into 5 groups: placebo, positive control group (50 mg/kg metformin), and three groups with different dosages of conglutin gamma (10.5, 21.0, and 42.0 mg). 30 minutes before the glucose overloading experiment (2 g/kg) the treatment was administrated. At 0, 30, 60, and 90 min from glucose administration all rats received a dose of 50 mg/kg Na thiopental. Another part of the study concerned humans (a placebo-controlled study) and was performed on 15 healthy adult participants. By 7 weeks, participants were offered either one of three doses of powder from Lupin plant extract (750, 1500, and 3000 mg = 157.3, 315, and 630 mg conglutin gamma) or placebo 30 minutes before a meal consisting of 85 g of white rice (75 g carbohydrate). A study on rats showed a statistically significant correlation between the dosage of powder extract and blood glucose level. According to the dosage level of glucose, it was reduced by 14%, 42%, and 64%. The effect obtained after administration of the highest dosage was not different from the effect of the administration of 50 mg/kg metformin. The results of human studies confirmed the hypoglycemic properties of conglutin gamma. Compared to the control group, after three doses in 60 minutes lowered glucose levels averaged 81%. In 90 minutes, the 750 mg dosage caused an increase of the glucose level to 83% when compared to the placebo, but 1500 mg increased about 61% and the dosage of 3000 mg increased the glucose level about 71% compared to the placebo group. As a result of measurements of the areas under the curve glucose was significantly decreased by dosages of 1500 mg of 75% and 3000 mg of 79% compared to placebos. There were no significant differences in the level of serum insulin [37].

## 7. Bioavailability of Bean Active Components

The interest in active components included in food is caused predominantly by the results of epidemiological examinations. In order to establish unambiguous evidence of the effectiveness of these compounds, including polyphenols, in their role as disease prophilaxy, it is indispensable to determine their bioavailability and next their biological activity. Bioavailability of assorted phenol compounds varies a lot, and those which are mostly consumed in a staple diet do not always manifest positive bioavailability profiles [62].

The majority of the research tasks dealing with the influence of phenol compounds coming from food products were conducted under* in vitro* conditions or on animal models, considering concentrations higher than those consumed in an everyday diet. It should be noted that polyphenol profiles for the given product usually remain the same, whereas differences in concentrations occur, for example, in the case of Cabernet Sauvignon wine grapes in the Napa Valley (California, USA) in 1989. It contained 0.9 mg/L resveratrol, while in the year 1994 it contained 8.9 mg/L.

The main factors influencing polyphenol bioavailability are as follows: environmental factors (exposure to sun rays, maturity level), food product availability, cooking and heat treatment, homogenisation, lyophilisation, storage, and the presence of other compounds which suppress or enhance assimilation, compounds made with proteins or other polyphenols with similar absorption mechanisms, chemical structure, concentrations in food, the amounts consumed with food, enzyme activity, intestinal passage time, intestinal microflora composition, age, health condition, genetic factors, and physiological conditions [62].

Compounds found in leguminous plants, whose antidiabetic activity is supported by laboratory tests and on animal models (e.g. genistein, daidzein, alpha-amylase, and alpha-glucosidase inhibitors) display low bioavailability, genistein mainly due to its poor solubility in water. Alpha-amylase and alpha-glucosidase enzymes inhibitors lose their properties in heat treatment time [20, 60, 62].

## 8. Conclusion

1. Legumes should constitute a permanent dietary element in a balanced diet, especially with type 2 diabetic patients.
2. Legumes are a source of wholesome protein, alimentary fiber, and bioactive substances displaying antioxidant activity together with anti-inflammatory and antineoplastic properties.
3. The* in vitro* research using leguminous plants extracts confirmed their antidiabetic properties.
4. Leguminous plants should be employed in the promotion of a healthy lifestyle as a form of functional food.
5. More studies are required to estimate the potential role of the leguminous plants in therapy including cancer, inflammation, and coronary diseases.

## Supplementary Material

The table shows the results of In vivo human studies regarding active compounds from legumes conducted in the years 2004-2013. In most of these studies was observed an improvement of parameters such as blood glucose levels, insulin sensitivity, BMI, food intake, or other subjects.

## Figures and Tables

**Table 1:** The antidiabetic effect of the active compounds from legumes in research conducted in 2004–2014.

Authors	Type of study	Bioactive compounds	Glycaemia	Insulin	Body weight	Food intake	Other activities
Udani et al., 2004 [24]	*In vivo* Human double-blank test	1500 mg water extract of a common white bean/day	—	—	↓BMI	—	↓Triglycerides level

Udani and Singh, 2007 [25]	*In vivo* Human double-blank test	1000 mg fractionated white bean extract/day	—	—	↓BMI	—	—

Celleno et al., 2007 [26]	*In vivo* Human randomized, double-blind, placebo-controlled	445 mg Phase 2 + 0.5 mg of chromium picolinate	—	—	↓BMI	—	↓Body weight ↓Fat mass ↓WHR

Maruyama et al., 2008 [27]	*In vivo* Human double-blank test	Azuki juice 150 g ∗ 5/day	—	—	↓BMI	—	↓Triglycerides level

Maruyama et al., 2008 [27]	*In vitro*	100 mg Azuki bean extract	—	—	—	—	↓Activity alpha-glucosidase by 32.6%

Yao et al., 2008 [28]	*In vivo* Mice control group	Vitexin and isovitexin MBS (3 and 2 g/kg ∗ 11.68, 5.40 mg/g) MBSC (3 g/kg ∗ 15.22, 11.42 mg/g)	↓Blood glucose level	↑Insulin immunoreactivity	—	—	↓Plasma C-peptide, ↓Glucagon, ↓total cholesterol, ↓Triglycerides, ↓BUN

Choi et al., 2008 [29]	*In vivo* Mice control group	Genistein and daidzein 0.2/kg/day	↓Glucose level in blood	↑Insulin production	—	—	↓Triglycerides gradient in liver

Kim et al., 2008 [30]	*In vivo* Mice control group	5 g genistein/100 g diet	—	↑Insulin production ↑Insulin sensitivity	—	—	—

Cederroth et al., 2008 [31]	*In vivo* Mice control group	198 ppm daidzein and 286 ppm genistein/day	—	↑Insulin sensitivity	—	—	↓Lipid contents in adipocytes, ↑Phosphorylation of AMPK and acetyl-CoA carboxylase (ACC), ↑Expression of (PPR-l *γ*)

Fu and Liu, 2009 [32]	*In vitro* clone cells insulin secreting (INS-lE)	Genistein	—	↑Glucose-stimulated insulin secretion	—	—	—

Vinson et al., 2009 [33]	*In vivo* Human crossover, placebo-controlled	Phase 2 750 mg, 1500 mg	↓Blood glucose level	—	—	—	—

Udani et al., 2009 [34]	*In vivo* Human crosses, randomized study	Phase 2 1500, 2000, 3000 mg	↓Blood glucose level	—	—	—	—
Wu et al., 2010 [35]	*In vivo* Human double-blinded placebo-controlled study	Phase 2 2000 mg	—	—	↓BMI	—	No changes in WHR

Gobert et al., 2010 [36]	*In vivo* Human placebo-controlled group	88 mg isoflavones (genistein, daidzein, and glycitein)/day equol	No change in fasting and postprandial glucose or insulin levels	No change in indexes of insulin sensitivity and resistance	—	—	No change in HbA1c levels

Bertoglio et al., 2011 [37]	*In vivo* Rats placebo-controlled trial	*γ*-Conglutin 10.5 mg, 21 mg, and 42 mg	↓Glucose level in blood	—	—	—	—

Bertoglio et al., 2011 [37]	*In vivo* Human placebo-controlled trial	*γ*-Conglutin 157.5 mg, 315 mg, and 630 mg	↓Glucose level in blood	—	—	—	—

Dove et al., 2011 [38]	*In vivo* Human control group	22 g lupin protein	↓Postprandial glycaemia	—	—	—	—

Spadafranca et al., 2013[39]	*In vivo* Human double-blind, randomized, crossover study	Alpha-amylase inhibitor 6% ∗ 100 mg	↓Blood glucose level	↑Insulin level	—	↓Food intake	↓C-Peptide concentration in blood plasma ↓Ghrelin after a meal

Choo et al., 2012 [40]	*In vivo* Mice and rats control group	Vitexin 1, 3, and 15 mg 50, 100, and 200 mg Isovitexin 1, 3, and 15 mg 20, 50, and 100 mg	↓Postprandial glycaemia	—	—	—	—

Fu et al., 2012 [41]	*In vivo* Mice control group	Genistein 250 mg ∗ kg (−1)/day	↓Hyperglycaemia ↑Glucose tolerance—positive *β* cells of the islets	↑Insulin levels in the blood No change in insulin sensitivity ↑Number of insulin	No change	No change	—

Squadrito et al., 2013 [42]	*In vivo* Human placebo-controlled trial	Genistein 54 mg/day	↓Glucose levels	↓Insulin levels	No change	—	↓Total cholesterol and triglyceride ↑HDL levels ↓Systolic and diastolic BP ↓Visfatin and homocysteine levels

Kim and Lim, 2013 [43]	*In vivo* Mice control group	Genistein 0.1%, 0.025% in daily diet	0.025 ↓FBG		No change	↑Food intake	—

Loi et al., 2013 [44]	*In vivo* Rats control group	Alpha-amylase inhibitor/caffeoylquinic acid	↓Blood glucose level	—	—	↓Food intake	—

Park et al., 2013 [45]	*In vivo* Mice control group	Daidzein 10 mg ∗ kg body weight/day	↓Postprandial glycaemia	—	—	—	—
Yao and Ren, 2014 [46]	*In vivo* Rats control group	Extruded adzuki bean extract 200 mg	↓Blood glucose level	—	—	—	—

Capraro et al., 2014 [47]	*In vivo* Rats placebo-controlled trial	*γ*-conglutin 125 mg	↓Glucose level in blood	—	—	↓Food intake	—

## Abbreviations

ABE/EABE: Adzuki bean extract/extruded adzuki bean extract
ACC: Acetyl-CoA carboxylase
Alfa-AI1: a-Amylase inhibitor isoform 1
Alfa-AI2: a-Amylase inhibitor isoform 2
Alfa-AIL: a-Amylase inhibitor isoform like
AMPK: AMP-activated protein kinase
ATP: Adenozynotrifosforan
BMI: Body mass index
BUN: Blood urea nitrogen
CA: Concentrated Azuki
cAMP: Cyclic adenosine monophosphate
CCK: Cholecystokinin
CSE: * Cynara scolymus* extract
DMC: Diabetic mice control group
DMH/DMH-C: Diabetic mice height FBG/-control group
DMMH/DMMH-C: Diabetic mice medium hight FBG/-control group
EGCG: Epigallocatechingallate
EtOAc: Ethylacetate
FBG: Fasting blood glucose
GI: Glycemic index
GLUT4: Insulin-regulated glucose transporter
GPT: Glutamate pyruvate transaminase
GSIS: Glucose-stimulated insulin secretion
INS-1E: Clone cells insulin secretion
IRS-1: Insulin receptor substrate 1
K_ATP_: ATP-sensitive potassium
MBS/MBSC: Mung bean sprouts extract/mung bean seed coat extract
mTOR: Pathway mammalian target of rapamycin
PEPCK: Phosphoenolpyruvate carboxykinase
PGC-1*γ*: Peroxisome proliferator-activated receptor *γ* coactivator
PHA: Phytohaemagglutinin
PPAR*α*: Peroxisome proliferator-activated receptor alpha
PPR1^*γ*^: Peroxisome proliferator-activated receptor gamma coactivator
PVE: * Phaseolus vulgaris* extract
ROS: Reactive oxygen species
S6K1: Ribosomal protein S6 kinase beta-1
SHARP 2: Hypoxia-regulated transcription factor
SPI/MPI: Diet containing soy protein/diet containing milk protein.
