# Supplementary material for: The Active Role of Leguminous Plant Components in Type 2 Diabetes
Source: Evid Based Complement Alternat Med. 2014 Mar 11;2014:293961. doi: 10.1155/2014/293961 (PMC3967837; doi:10.1155/2014/293961)
Supplement: Supplementary file 1 — The table shows the results of In vivo human studies regarding active compounds from legumes conducted in the years 2004-2013. In most of these studies was observed an improvement of parameters such as blood glucose levels, insulin sensitivity, BMI, food intake, or other subjects. [file 293961.f1.pdf]

Supplementary table 1. The antidiabetic effect of the active compounds from legumes in *In vivo* human studies

Autors	Type of study	Bioactive compounds	Glycaemia	Insulin	Body weight	Food Intake	Other activity
Udani et al., 2004 [10]	*In vivo* Human double blank test	1500 mg water extract of a common white bean/day	-	-	↓BMI	-	↓ Triglycerides level
Udani and Singh, 2007 [11]	*In vivo* Human double blank test	1000mg fractionated white bean extract/day	-	-	↓ BMI	-	-
Celleno et al., 2007 [12]	*In vivo* Human randomiz ed, double-blind, placebo-controlled	445 mg Phase 2 + 0.5 mg of chromium picolinate	-	-	↓BMI	-	↓body weight ↓fat mass ↓WHR
Maruyam a et al., 2008 [13]	*In vivo* Human double blank test	Azuki juice 150g*5/day	-	-	↓ BMI	-	↓ Triglycerides level
Vinson et al., 2009 [19]	*In vivo* Human cross-over,	Phase 2 750mg, 1500mg	↓ blood glucose level	-	-	-	-

	placebo-controlled						
Udani et al., 2009 [20]	*In vivo* Human crosses, randomized study	Phase 2 1500, 2000, 3000mg	↓ blood glucose level	-	-	-	-
Wu et al., 2010 [21]	*In vivo* Human double blinded placebo-controlled study	Phase 2 2000 mg	-	-	↓BMI	-	No changes WHR
Gobert et al., 2010 [22]	*In vivo* Human placebo-controlled group	88 mg isoflavones (genistein, daidzein, glycitein)/ day equol	No change fasting and postprandial glucose or insulin levels	No change indexes of insulin sensitivity and resistance	-	-	No change HbA1c levels
Bertoglio et al., 2011 [23]	*In vivo* Human placebo controlled trial	γ-conglutin 157. 5mg, 315mg, 630mg	↓Glucose level in blood	-	-	-	-
Dove et al., 2011 [24]	*In vivo* Human Control group	22 g lupin protein	↓ Postprandial glycaemia	-	-	-	-
Spadafranca et al., 2013 [25]	*In vivo* Human double-blind, randomized, crossover study	alpha-amylase inhibitor 6%*100mg	↓blood glucose level	↑Insulin level	-	↓food intake	↓C -peptide concentration in blood plasma ↓ghrelin after a meal
Squadrito et al., 2013 [28]	*In vivo* Human placebo controlled trial	Genistein 54 mg / day	↓Glucose levels	↓Insulin levels	No change	-	↓Total cholesterol and triglyceride ↑HDL levels ↓Systolic and diastolic BP ↓visfatin and homocysteine levels
